# Evaluation of Metallo-β-Lactamase Susceptibility Testing in a Physiologic Medium

**DOI:** 10.1128/Spectrum.01670-21

**Published:** 2021-11-24

**Authors:** Tomefa E. Asempa, Hannah Bajor, Jessica H. Mullins, Janice Hartnett, David P. Nicolau

**Affiliations:** a Center for Anti-Infective Research and Development, Hartford Hospital, Hartford, Connecticut, USA; b Women’s Ambulatory Health Services, Hartford Hospital, Hartford, Connecticut, USA; c Division of Infectious Diseases, Hartford Hospital, Hartford, Connecticut, USA; National University Hospital

**Keywords:** β-lactamases, β-lactams, metalloenzymes

## Abstract

Research in identifying alternative growth media that better mimic host conditions is gaining ground. Relative to nutrient-rich Mueller-Hinton broth (MHB), data on the influence of physiologic or host-mimicking media on metallo-β-lactamase (MBL) resistance are lacking. The objective was to evaluate meropenem susceptibility against clinical and engineered MBL-harboring *Enterobacterales* strains in a physiologic medium (urine). Antimicrobial susceptibility testing (AST) by broth microdilution was conducted with a wild-type Klebsiella pneumoniae strain and two engineered isogenic variants harboring K. pneumoniae carbapenemase 2 (KPC-2) or New Delhi MBL 1 (NDM-1), as well as two clinical K. pneumoniae isolates (harboring NDM-1 and VIM-1). MICs were determined in conventional cation-adjusted MHB (caMHB) and sterile-filtered urine samples (18 patients). All KPC- and MBL-harboring isolates were meropenem resistant (MICs of ≥16 mg/liter) in caMHB. AST of the KPC isolate in urine resulted in 50% (9/18 urine samples) essential agreement (i.e., within ±1 dilution, relative to the caMHB MIC), highlighting challenges with the use of urine as a medium capable of supporting AST. In the 9 AST-viable urine samples, meropenem MICs were 2- to 9-fold lower than that in caMHB (MIC of 32 mg/liter) among MBL-harboring isolates. Zinc concentrations determined by inductively coupled plasma mass spectrometry averaged 1.25 mg/liter and ranged from 0.12 to 1.14 mg/liter in caMHB and 18 urine samples, respectively. The full extent of MBL-mediated resistance among K. pneumoniae isolates appears to be attenuated in urine. Factors influencing free bioactive zinc levels warrant further investigation.

**IMPORTANCE** Studies assessing antibiotic susceptibility profiles in nonconventional media are lacking. MBL-mediated resistance has come under scrutiny due to the dependence on extracellular zinc concentrations, which makes the choice of testing medium influential for β-lactam MICs. This study explores human urine as a physiologically relevant matrix with which susceptibility profiles of MBL-harboring isolates can be assessed, relative to conventional broth.

## OBSERVATION

The characterization of antimicrobial resistance through antimicrobial susceptibility testing (AST) is an important part of local/global isolate surveillance, selection of therapeutic agents for patients, and discovery of new agents (1). Testing is routinely performed in conventional growth media such as Mueller-Hinton broth (MHB), but discussion about the development of host-mimicking media as alternative testing media is gaining traction. This is a result of recent studies highlighting the limitations of MHB in appropriately recapitulating host (*in vivo*) conditions, given its nonphysiologic ion concentrations (besides calcium and magnesium) and its nutrient-rich component (beef extract) (2–7).

It is well known that interactions between host and pathogen can affect the efficacy of clinical therapeutics. The magnitude of several resistance mechanisms for both Gram-positive and Gram-negative isolates has been shown to be affected by physiochemical factors in the pathogen environment (8–10). For example, in a study to investigate the potential benefits of macrolides administered to patients with cystic fibrosis, Buyck and colleagues elegantly showed that, despite elevated MICs (>512 mg/liter) in cation-adjusted MHB (caMHB), azithromycin demonstrated antimicrobial activity against azithromycin-resistant Pseudomonas aeruginosa in eukaryotic medium (RPMI 1640 medium) and mouse bronchoalveolar lavage (BAL) fluid (9). Briefly, reverse transcription PCR studies showed downregulation of *oprM* by azithromycin in RPMI 1640 medium, and outer membrane permeability was 3 to 4.5 times higher in RPMI 1640 medium and BAL fluid, compared with caMHB (9). Similarly, we have shown that variability in zinc concentrations of caMHB impacts meropenem resistance among metallo-β-lactamase (MBL)-harboring *Enterobacterales* strains (7). Other studies have also shown that environmental cues can regulate expression of virulence factors among pathogens (11). Taken together, these results indicate that continuous interrogation of resistance mechanisms in alternative or physiologic media versus conventional media may provide insights into variable resistance expression and susceptibility profiles. In the current study, we sought to compare meropenem susceptibility profiles of MBL-harboring *Enterobacterales* strains determined in caMHB and biologic urine as a physiologic medium.

Urine sample collection was carried out at the Women’s Ambulatory Health Services, Hartford Hospital, after patients signed a Hartford HealthCare institutional review board (IRB)-approved informed consent form (form HHC-2020-0167). Patients (≥18 years of age) were receiving routine obstetric or gynecologic care, were not taking antibiotics, and did not have a urinary tract infection (as confirmed by routine urinalysis). Urine samples were frozen immediately at −80°C. Samples were thawed and sterile filtered (Whatman filters, 0.22-µm pore size) prior to preparation of microdilution trays (100% urine).

The bacterial strains are listed in Table 1 and included a wild-type (WT) Klebsiella pneumoniae ATCC 10031 strain and two engineered isogenic variants, harboring New Delhi MBL (NDM-1) (WT-NDM) or K. pneumoniae carbapenemase 2 (KPC-2) (WT-KPC) (12), as well as two clinical K. pneumoniae isolates (harboring NDM-1 and VIM-1). AST via microdilution in duplicate or singlet (depending on urine volume) was performed according to CLSI methods (13). AST was conducted in (i) 18 individual urine samples, (ii) 2 pooled samples (each pooled from 3 unique patient samples, and (iii) caMHB (BBL lot 9324795; Becton, Dickinson and Co.). Bacteria inocula were made in the respective media. Analytical-grade meropenem (lot LRAB7853; Sigma-Aldrich) was utilized for AST (MIC tray range, 0.06 to 64 mg/ml). P. aeruginosa ATCC 27853 was used as a meropenem quality control isolate, as defined by the CLSI (14).

**TABLE 1:** Medium characteristics and meropenem MICs of clinical and engineered MBL-producing isolates in urine samples (*n* = 18 patients)

Medium	Total zinc concn (mg/liter)	pH^*a*^	Meropenem MIC (mg/liter) for:				
			WT	WT-KPC-2	WT-NDM-1	NDM-1^*b*^	VIM-1^*c*^
caMHB	1.25	7.2	≤0.06	16	64	32	64
Urine sample 1	1.14	7.5	≤0.06	≤0.06	1	1	0.25
Urine sample 2	0.8	7.5	≤0.06	0.25	0.25	1–0.5	No growth
Urine sample 3^*d*^	0.93	6	0.125	8^*e*^	0.125	0.25	≤0.06
Urine sample 4	0.58	6	≤0.06	2	≤0.06	0.125	≤0.06
Urine sample 5	0.56	6	≤0.06	2	0.125	≤0.06	1
Urine sample 6^*d*^	0.46	7	≤0.06	16^*e*^	8	4	2
Urine sample 7	0.28	8	No growth	No growth	No growth	1	1
Urine sample 8^*d*^	0.26	5.5	≤0.06	16^*e*^	0.125	0.25	≤0.06
Urine sample 9^*d*^	0.12	7	≤0.06	8^*e*^	8	2	0.5
Urine sample 10^*d*^	0.59	6	≤0.06	8^*e*^	0.25	0.125	0.125 to ≤0.06
Urine sample 11	0.8	6	≤0.06	2	≤0.06	≤0.06	≤0.06
Urine sample 12	0.42	7	0.125	1	0.25	0.25	0.125
Urine sample 13^*d*^	0.31	6	≤0.06	8^*e*^	≤0.06	≤0.06	≤0.06
Urine sample 14	0.15	6	≤0.06	1	≤0.06	≤0.06	≤0.06
Urine sample 15^*d*^	0.64	7	≤0.06	16^*e*^	2	16	0.25
Urine sample 16	0.18	6.5	≤0.06	4	≤0.06	≤0.06	≤0.06
Urine sample 17^*d*^	0.32	6.5	≤0.06	8^*e*^	≤0.06	≤0.06	≤0.06
Urine sample 18^*d*^	0.59	6.5	≤0.06	16^*e*^	0.125	0.125	≤0.06
Urine pool 1^*d*^^,^^*f*^	0.56	ND	≤0.06	16^*e*^	0.125	0.25–0.5	0.25
Urine pool 2^*d*^^,^^*f*^	0.52	ND	≤0.06	16^*e*^	4	2	0.5

a Urine pH was obtained from urinalysis.

b KP 593 (harbors NDM-1, SHV-11, CTX-M-15, and OXA-1).

c KP 470 (harbors VIM-1).

d MIC values with ≥2-fold dilution changes among MBL-harboring isolates in AST-viable urine.

e Urine samples that demonstrated KPC MIC essential agreement between urine and caMHB.

f ND, not determined. Urine pool 1 was pooled from patient samples 3, 13, and 18, and urine pool 2 was pooled from patient samples 1, 9, and 16.

Zinc concentrations were measured by inductively coupled plasma mass spectrometry (ICP-MS) using a 7500 CE instrument (Agilent Technologies, USA) with a lower limit of detection of 0.002 mg/liter. Protein binding was performed for all urine samples using a centrifugal filter (Ultracel 3K [Amicon Ultra 0.5mL; Lot no. R0NB38503]), which was centrifuged at 14,000 × *g* for 30 min.

caMHB-derived meropenem MIC values served as the reference standard to which MIC values determined in urine were compared. The KPC isolate (WT-KPC) was included as a non-MBL-expressing control isolate. Meropenem susceptibility results were interpreted according to CLSI guidelines (14). Given the well-described challenges with microbial growth in urine, a urine sample was considered viable from an AST perspective only if the meropenem MIC of the KPC isolate determined in urine was within ±1 doubling dilution of the MIC determined in caMHB, i.e., essential agreement.

All MBL- and KPC-harboring *Enterobacteriaceae* demonstrated *in vitro* resistance to meropenem in caMHB (MIC range, 16 to 64 mg/liter). Approximately 50 ml of midstream urine was collected from 18 patients. Of the 18 urine samples tested as susceptibility media, 9 were deemed suitable, given essential agreement between the MICs generated in urine versus caMHB for the KPC-harboring isolate. Meropenem MICs tested with these 9 AST-viable urine samples were predominantly within the susceptible range (i.e., 2- to 9-fold reduction), relative to MICs generated in caMHB (meropenem MIC of >32 mg/liter) for both MBL-harboring engineered strains and clinical MBL-harboring isolates (Table 1). A similar observation was made with pooled urine (i.e., 4- to 8-fold reduction in meropenem MIC values).

Samples of caMHB and urine utilized in the susceptibility studies were assayed for zinc concentration. The mean zinc concentration in caMHB was 1.25 mg/liter, while the zinc concentration in urine ranged from 0.12 to 1.14 mg/liter (Table 1). Free zinc concentrations ranged from 61% to 100% (median, 91.5%) of total concentrations across the urine samples.

MHB, which was developed in the 1940s as a nutrient-rich and serum-free medium to support the growth of meningococci and gonococci, now serves as a growth medium for a large variety of nonfastidious bacterial isolates and has become an essential component of AST (14, 15). We recently reported that variations in both the cationic (specifically, supraphysiologic zinc concentrations) and noncationic contents of commercially available caMHB can impact β-lactam susceptibility testing of MBL-harboring *Enterobacterales* strains (7). In another study, the unexpected activity of β-lactams against β-lactam-resistant MBL-harboring isolates in a murine infection model suggested that the low zinc concentrations found *in vivo* as a result of protein binding and nutritional immunity (cation sequestration during infection) might be attenuating MBL hydrolytic activity, relative to the supraphysiologic zinc concentrations found in caMHB (6, 16–18). The availability of nutrients in MHB has been shown to be related to MIC, with a lower nutrient availability affecting pharmacodynamic characteristics through a lower bacterial growth rate and higher kill rate, as demonstrated in a study by Mouton (5, 19). Noting the abundance of nutrients *in vitro* versus *in vivo*, Mouton suggested that a nutritional factor should be considered when translating *in vitro* results to *in vivo* pharmacodynamics (19).

Unsurprisingly, not all of the urine samples were capable of supporting bacterial growth, which corroborates observations by others that variations in urine chemical and nutrient composition, including glucose, can influence microbial growth (20–22). Furthermore, in 8 of the 18 urine samples, AST of the KPC-harboring isolate did not result in comparable MICs, relative to MIC values generated in caMHB, likely due to the aforementioned composition factors. Notably, pH measurements were within the normal range and did not appear to explain variability in bacterial growth or essential agreement. Among the 9 AST-viable urine samples remaining, meropenem MICs for MBL-harboring isolates were severalfold lower than MICs generated in caMHB regardless of urine zinc content, suggesting that free zinc uptake was not optimal. This observation is in contrast to an MBL-harboring isolate study that showed no change in meropenem MICs in pooled urine, relative to those in caMHB (23). Because the MIC reduction was limited to isolates harboring an MBL and not KPC, we hypothesize that the attenuated MBL activity is likely related to zinc homeostasis. These results highlight gaps in our understanding of the mechanisms and factors required to mediate MBL resistance as it pertains to protein binding in humans, as well as different extracellular conditions besides conventional testing media. While zinc plays a central role in MBL enzyme folding and β-lactam hydrolysis (24), the interplay of other physiochemical factors is seldom addressed. Indeed, it has yet to be elucidated what molecules besides protein (i.e., albumin) affect zinc buffering and uptake across the cell membrane (25, 26).

We would like to reiterate challenges involved in conducting studies involving patients. In fact, more than 40 patients were enrolled, but low urine volumes or contaminated urine samples precluded their use in subsequent AST studies. We also recognize the importance of conducting broth microdilution tests in triplicate, as performed in our previous studies (6, 7). We deemed it important to determine the full range of meropenem MICs (i.e., a broth microdilution tray that spans 11 dilutions [0.06 to 64 mg/liter]) to be able to ascertain any changes in MIC for each isolate. It was also important that we included an experimental control genotype (KPC isolate). The volume of urine per patient required to ensure that we could incorporate these two study design elements limited the total number of MBL isolates we could run; therefore, future studies utilizing individual (i.e., not pooled) urine samples should look to obtain >100 ml per patient. The bacterial species evaluated were also limited to K. pneumoniae isolates only. Nonetheless, the large number of individual urine samples, as well as inclusion of engineered and clinical isolates, strengthens the study design. Besides assessing pH as a variable, characterizing the composition of each urine sample for other cationic and chemical composition features, including osmolality, was beyond the scope of this study and warrants further investigation.

Data from this study suggest that the full extent of MBL-mediated resistance among K. pneumoniae isolates is attenuated in urine. Progress in the field of AST is based on an iterative process in which microbiologic research informs clinical application, which then provides outcome data to optimize subsequent testing methods. Therefore, more research is needed to further our understanding of how extracellular zinc modulates MBL activity and what physiochemical factors buffer extracellular zinc ions. Ultimately, we should strive to develop an AST medium that better reflects physiologic conditions in order to uncover the therapeutic potential of existing and preclinical-stage antimicrobials for all clinically relevant pathogens and not just MBL-harboring isolates.
